# Recurrence following percutaneous exclusion of giant coronary pseudoaneurysm: a case report

**DOI:** 10.1186/s43044-024-00546-7

**Published:** 2024-09-03

**Authors:** Saibal Mukhopadhyay, Jamal Yusuf, Ankur Gautam, Sanjeev Kathuria, Vishal Batra

**Affiliations:** Department of Cardiology, GB Pant Hospital, Academic Block, First Floor, Room No. 129, 1, Jawaharlal Nehru Marg, 64 Khamba, Raj Ghat, New Delhi, Delhi 110002 India

**Keywords:** Coronary artery aneurysm, Pseudoaneurysm, Stent grafts, Guide extension catheter, Optical coherence tomography, Percutaneous coronary intervention

## Abstract

**Background:**

Emergence of coronary giant pseudoaneurysm (PSA) after stent implantation is potentially catastrophic and may end up with life threatening complications if not managed promptly. There is scarcity of data in existing literature with respect to guidelines on the management of coronary PSA following stent implantation. We report the recurrence of coronary PSA following initial percutaneous management of a giant coronary PSA using multiple stent grafts.

**Case presentation:**

A 38-year-old male who underwent primary angioplasty of the right coronary artery (RCA) about a month back, presented with dull aching precordial chest pain for the last 15 days. A repeat coronary angiography revealed giant coronary PSA in proximal to mid RCA. Considering the significantly large size of the coronary PSA with symptoms of impending rupture, the giant coronary PSA was successfully excluded by implanting three sequentially coronary stent grafts. However, after one and a half months, the patient again presented with a similar kind of dull aching chest pain. We found a recurrence of coronary PSA in a segment of the coronary artery distal to the portion excluded by stent grafts. This recurrent coronary PSA was once again successfully excluded by redeploying two more stent grafts with the help of a guide extension catheter.

**Conclusions:**

In this case, vessel wall injury as a result of aggressive post dilatation using an oversized balloon during the index procedure was the contributor to the giant coronary PSA formation. It usually appears early after the index procedure (within 4 weeks). Though the usual strategy used to exclude coronary aneurysm is by using the minimal number of stent grafts (due to the inherent increased risk of restenosis/thrombosis in stent grafts) in post angioplasty traumatic aneurysm it is prudent to exclude the entire damaged artery by placing stent grafts to prevent recurrence in segments with even minimal dilatation on initial evaluation.

**Supplementary Information:**

The online version contains supplementary material available at 10.1186/s43044-024-00546-7.

## Background

Occurrence of coronary artery pseudoaneurysm (PSA) is a rare and under-diagnosed complication following implantation of drug eluting stent (DES). A coronary artery aneurysm is defined as localized dilatation of the coronary artery exceeding 1.5 times of reference vessel diameter and if the diameter exceeds the reference vessel diameter by > 4 times it is referred to as a giant coronary aneurysm [1, 2]. Giant coronary PSAs often present with non-specific symptoms but can lead to fatal complications like rupture, myocardial infarction and sudden death [3, 4]. Hence, timely diagnosis and early intervention are essential to improve the outcome of patients with giant coronary PSA. We report recurrent formation of a coronary PSA following successful exclusion of a giant coronary PSA by three stent grafts necessitating repeat intervention and treatment by additional stent graft.

## Case presentation

A 38-year-old hypertensive, smoker and non-diabetic male underwent primary percutaneous coronary intervention (PCI) of the right coronary artery (RCA) about a month back in a peripheral center. He presented with dull aching constant precordial chest pain, fifteen days following the procedure. On presentation, he was afebrile with no abnormality detected on cardiovascular examinations. The 12 lead electrocardiogram (ECG) did not show any fresh changes compared to old ECGs and the cardiac biomarkers (CK-MB and troponins) were also normal. The left ventricular ejection fraction assessed by 2D echocardiography was around 60% without any regional wall motion abnormalities.

Records of angioplasty revealed that the patient had undergone successful primary PCI of RCA. Two sequential Zotarolimus eluting stents Resolute Onyx (Medtronic, CA, USA) of size 3 × 24 mm and 3.5 × 32 mm were deployed at 12 atm pressure from distal to proximal RCA. Post dilatation was done using 3.25 × 12 mm and 4 × 12 mm non- compliant Sprinter balloons (Medtronic, Minneapolis, USA) at high pressure (18 atm pressure) resulting in thrombolysis in myocardial infarction (TIMI)-3 flow.

**Fig. 1:**
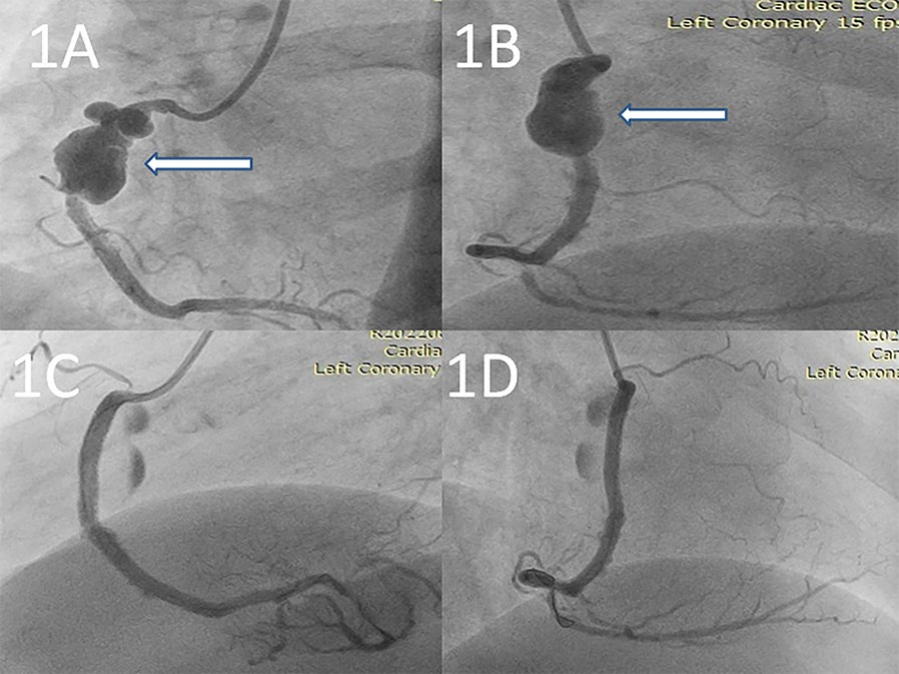
**A**, **B** Coronary angiogram of right coronary artery in Left anterior oblique (LAO) and right anterior oblique (RAO) view, respectively, shows giant coronary PSA (arrow denotes). **C**, **D** Coronary angiogram shows complete exclusion of giant coronary PSA after implanting stent grafts in LAO and RAO view, respectively

Check angiogram revealed a giant coronary PSA in the proximal and mid portion of RCA stents (Fig. 1A and B) (videos 1 and 2). Considering the significantly large size of the coronary PSA with symptoms of impending rupture, the decision of urgent percutaneous exclusion using coronary stent grafts was undertaken. Thereafter, three coronary stent grafts [2 Graftmaster (Abbott Vascular, Santa Clara, CA, USA) (2.8 × 16 mm, 3.5 × 16 mm) and PK Papyrus (Biotronik AG, Bülach, Switzerland) 3.5 × 20 mm] were placed sequentially from mid to proximal RCA to exclude the coronary PSA successfully (Fig. 1C and D) (video 3) with TIMI-3 flow in RCA. He was discharged on day 5 of the procedure on medications comprising of dual antiplatelet therapy (aspirin 75 mg once a day and ticagrelor 90 mg twice a day), high intensity statins (rosuvastatin 40 mg/day) and bisoprolol 5 mg/day.

**Fig. 2:**
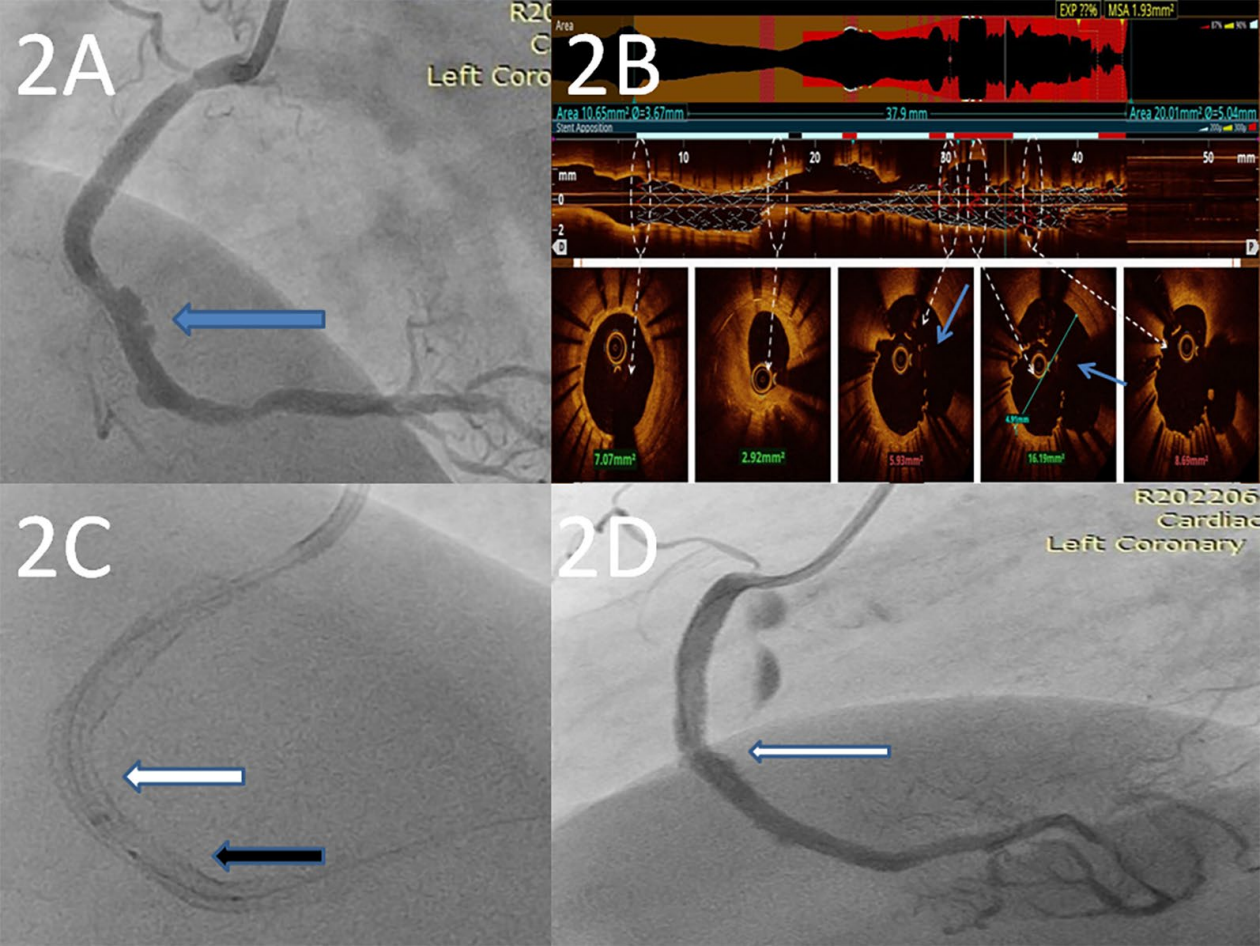
**A** Repeat coronary angiogram shows recurrence of coronary PSA distal to the deployed stent grafts (Arrow denotes). **B** OCT run of RCA shows coronary PSA (arrow denotes). **C** The black arrow denotes the guide extension catheter through which stent graft (white arrow) was delivered. **D** Arrow shows mild dilatation of the artery distal to the deployed stent grafts in the initial angiogram

After one and a half months of the procedure, the patient again presented with a similar complaint of dull aching precordial chest pain. A repeat coronary angiogram revealed coronary PSA of 1.2 × 1.2 cm just distal to the previously implanted stent grafts (Fig. 2A) (video 4). In view of constant symptoms, we decided to exclude this aneurysmal segment by placing coronary stent grafts. We also did a pre-procedural optical coherence tomography (OCT) to delineate the coronary PSA and extent of medial damage (Fig. 2B). However, negotiating a stent graft through previously placed stent grafts was difficult. Hence, we delivered a PK Papyrus stent graft (3 × 20 mm) through a 6F Guidezilla™ (Boston Scientific, Marlborough, MA) guide extension catheter (Fig. 2C). Further, after placement of the only available PK Papyrus stent graft distally to exclude the PSA segment, a small segment of the PSA segment remained uncovered proximally and needed exclusion by an additional stent graft. Due to the non-availability of another PK Papyrus stent graft, we planned to exclude the remaining uncovered segment by a Graftmaster stent graft. We needed a 7F guide extension catheter to deliver the stent graft as the higher profile Graftmaster stent cannot be passed through a 6F guide extension catheter. A Graftmaster stent graft (2.8 × 16) mm was delivered through a 7F Guidezilla™ guide extension catheter resulting in the complete exclusion of the coronary PSA. The patient was discharged successfully on day 3 and at six months follow-up, the patient remained asymptomatic.

## Discussion

Coronary PSA following PCI is rare, with a reported incidence of 0.3–6.0%; and giant coronary PSA is extremely rare with a reported incidence of 0.02% [1]. Giant coronary PSA often involves RCA adjacent to the right atrium, as the adjacent area of the atrium is weak that facilitates the development and expansion of the aneurysm [5].

Three different types of coronary PSA after PCI with stent implantation have been described [1] (Table 1). Type-I coronary PSA exhibits rapid early growth and it manifests within four weeks of the procedure. Oversized balloons or high-pressure balloon inflation causing medial disruption and weakening of the vessel wall leading to coronary PSA formation [6, 7]. Our case was a type-I coronary PSA secondary to post dilatation of the stents with oversized balloons at high pressure. Type-II coronary PSA has a sub-acute to chronic presentation and is usually detected 6 months after the procedure. A polymer, drug or metallic component of stents can induce chronic local hypersensitivity reaction that results in weakening of the arterial wall with subsequent dilatation [8]. The third subtype is mycotic or infectious in etiology (type-III coronary PSA) which presents with fever and systemic manifestations of bacteremia [9].

**Table 1:** Mechanism related to coronary aneurysm formation following DES implantation

Type-I coronary PSA (Mechanical injury of arterial wall)
Using oversized balloons/ stents
High-pressure balloon/stent inflation
Ablative techniques excisional atherectomy, laser angioplasty
Type-II coronary PSA
Polymer related hypersensitivity reaction or vasculitis
Incomplete endothelization due to local anti-proliferative drugs
Late stent malapposition
Type-III coronary PSA
Stent site infection

*DES* drug eluting stent, *PSA* pseudoaneurysm

Considering the recurrence of the coronary PSA, we did an attentive reassessment of the initial angiogram and found that there was mild dilatation of the artery distal to the deployed stent grafts (Fig. 2D). There was progressive dilatation of this damaged segment leading to recurrence. We suggest that in patients with type-1 coronary PSA, the entire segment of artery with evidence of medial damage should be excluded by placing stent grafts to prevent recurrence rather than targeting the maximally dilated part of the PSA.

In such cases, intravascular imaging should always be undertaken to document the extent of medial damage and its exclusion by placement of stent grafts. In our patient, during the initial procedure which was done on an urgent basis imaging could not be done due to the non-availability of the imaging facility. Secondly, during the re-intervention we faced technical challenges. As stent grafts are less trackable, we needed a 6F guide extension catheter [internal diameter (ID) 1.45 mm] to deliver a PK Papyrus stent graft (crimped profile 1.19 mm) distal to the previously placed stent grafts. As only one PK Papyrus stent graft was available, we realized that to pass a higher profile Graftmaster (a single graft sandwiched between 2 stents) we would require a 7F guide extension catheter (ID of 1.60 mm) which can accommodate a Graft master stent (crimped profile 1.57 mm) (Table 2).

**Table 2:** Size of available stent grafts (crimped profile) and their compatibility with guide extension catheter

ID of guide extension catheter (Guidzilla)	ED of PK Papyrus stent (crimped profile)—1.19 mm	ED of Graft master stent (crimped profile)—1.57 mm
6F-0.057″ (1.45 mm)	Compatible	Non-compatible
7F-0.063′ (1.60 mm)	Compatible	Compatible

*ID *internal diameter, *ED* external diameter

In the present case, we realized that even a small dilatation in a vessel which had been damaged initially can later develop into a clinically significant aneurysm. Hence, we decide that intravascular imaging (intravascular ultrasound or OCT) should be done during the initial presentation to delineate the extent of medial damage and the entire segment of the artery with medial damage irrespective of the degree of dilatation should be excluded by the placement of stent grafts to prevent future recurrence.

## Conclusions

A development of a giant coronary PSA following implantation of DES is a rare entity but may cause a serious catastrophe if not managed promptly. We recommend total length of the damaged artery (even if non-aneurysmal) to be treated and excluded by stent grafts to prevent future recurrence.

## Supplementary Information

Supplementary Video 1. Coronary angiogram in LAO view Shows a giant coronary PSA in the proximal and mid portion of RCA stents.

Supplementary Video 2. Coronary angiogram in lateral view Shows a giant coronary PSA in the proximal and mid portion of RCA stents.

Supplementary Video 3. Coronary angiogram shows complete exclusion of giant coronary PSA after implanting three sequential stent grafts.

Supplementary Video 4. Repeat coronary angiogram shows a recurrence of coronary PSA just distal to the previously implanted stent grafts.

## Data Availability

All data generated or analyzed during this study are included in this published article (and its supplementary information files).

## Abbreviations

PSA: Pseudoaneurysm
DES: Drug eluting stent
PCI: Percutaneous coronary intervention
RCA: Right coronary artery
ECG: Electrocardiogram
TIMI: Thrombolysis in myocardial infarction
OCT: Optical coherence tomography
ID: Internal diameter
